# Supercritical fluid extract of *Angelica sinensis* promotes the anti-colorectal cancer effect of oxaliplatin

**DOI:** 10.3389/fphar.2022.1007623

**Published:** 2022-11-04

**Authors:** Doudou Hao, Jia Liu, Ziyou Guo, Jiajia Chen, Tingting Li, Xin Li, Kai Mei, Lingmin Wang, Xinyi Wang, Qing Wu

**Affiliations:** School of Chinese Materia Medica, Beijing University of Chinese Medicine, Beijing, China

**Keywords:** *Angelica sinensis*, oxaliplatin, colorectal cancer, traditional Chinese medicine, combination therapy

## Abstract

Oxaliplatin-based chemotherapy regimens are recommended for patients with advanced colorectal cancer (CRC). However, oxaliplatin (OXA) can cause toxic side effects at the recommended dosage. Therefore, it is necessary to find new drug candidates that can synergize with OXA and thereby lower the OXA dose while still maintaining its efficacy. *Angelica sinensis* is a common drug in traditional Chinese medicine and has demonstrated a significant anti-CRC effect in modern pharmacological studies. The active ingredients in *Angelica sinensis* can be effectively extracted by a supercritical fluid extract. In this study, the supercritical fluid extract of *Angelica sinensis* (A-SFE) was obtained by a stable extraction process and was chemically characterized by GC/MS. The anti-cancer effect of A-SFE when applied individually was explored *in vitro* through MTT, scratch, and Transwell assay. The effect of A-SFE on CRC cells under the influence of tumor-associated macrophages (TAMs) was explored by a co-culture model. The results showed that A-SFE could inhibit the viability, metastasis, and invasion of HCT116 cells, especially under the influence of TAMs. When 20–100 μg/ml of A-SFE and 8–64 μg/ml of OXA were used in combination in HCT116 cells, synergistic or additive effects were shown in different concentration combinations. The CT26 syngeneic mouse model was used to explore the anti-CRC effect of OXA combined with A-SFE *in vivo*. The tumor volume, expression levels of Ki67, MMP9, and CD206 in the OXA + A-SFE group were less than those in the OXA group. In conclusion, A-SFE has the potential to become an adjuvant drug for OXA in the treatment of CRC, which provides new strategies for anti-colorectal cancer research.

## Introduction

Colorectal cancer (CRC) has the third highest incidence and the second highest mortality worldwide (Sung et al., 2021). Currently, adjuvant chemotherapy following surgery is recommended to most CRC patients (Smith and Garcia-Aguilar, 2015). In particular, oxaliplatin-based chemotherapy, such as mFOLFOX6 and CapeOX, is the first line choice of chemotherapy options for patients with advanced or metastatic CRC in current treatment (Benson et al., 2021). However, it has been reported that oxaliplatin (OXA) can cause neurotoxicity of the peripheral nervous system and other side effects, such as hearing loss, vomiting, and diarrhea, due to the high dose of clinical application (Oun et al., 2018). Therefore, researchers are actively searching for new drugs that can synergize with chemotherapy drugs to enhance the sensitivity of chemotherapy by regulating multiple metabolic pathways (Rejhova et al., 2018). Working in such a synergy, lower doses of chemotherapy combined with natural drugs can achieve the same effect as the original dose of chemotherapy. Combination therapy can reduce the dosage of conventional chemotherapeutic agents, thereby alleviating their toxicity and side effects.

It has been shown that natural drugs and the compounds derived from them exhibit prominent anti-cancer effects and may supplement current cancer treatment. Therefore, the combination of natural drugs and chemotherapy drugs has become a promising research topic to reduce the side effects of chemotherapy drugs and increase the sensitivity of chemotherapy. For example, cinnamaldehyde can increase the curative effect of OXA by synergistically promoting apoptosis, both *in vitro* and *in vivo* (Wu et al., 2019). Ursolic acid can enhance the therapeutic effect of OXA against CRC and minimize oxaliplatin-induced adverse events, both *in vitro* and *in vivo* (Shan et al., 2016). Aqueous extract of *Forsythia suspensa* fruits have shown an ameliorative effect on oxaliplatin-induced neurotoxicity *in vitro* and *in vivo*, which would reduce drug withdrawal due to side effects (Yi et al., 2019). Hence, it is of great significance to find ideal drug candidates with a synergistic effect on OXA from natural resources, and it may offer an optimized treatment regimen to CRC.

*Angelica sinensis* (named Danggui in Chinese) is the dried root of *Angelica sinensis* (Oliv.) Diels. As a common drug in traditional Chinese medicine, *Angelica sinensis* has been demonstrated a significant anti-colorectal cancer effect in modern pharmacological studies. The combination of Danggui Buxue Decoction, Fe, and rhEPO has shown anti-colorectal cancer activity *in vivo* (Shi et al., 2021). *Angelica sinensis* extract administration in either the initial or promotion stages of the Azoxymethane/Dextran sodium sulphate (AOM/DSS) mouse model reduced tumor incidence and high-grade intraepithelial neoplasia incidence (Zhao et al., 2017). The main active components of *Angelica sinensis*, especially the phthalide class, have also been reported to have antitumor and anti-metastasis activity (Long et al., 2012; Yin et al., 2013). Z-ligustilide inhibited the autophagy in tamoxifen-resistant breast cancer cells, and thereby enhanced the efficacy of tamoxifen therapy *in vitro* (Qi et al., 2017). Three main *Angelica sinensis* phthalides (i.e., Z-ligustilide, n-butylidenephthalide, and senkyunolide A) had anti-cancer potential. These three phthalides in combination with other ingredients in *Angelica sinensis* extract display a significant synergy leading to a stronger anti-cancer effect *in vitro* (Kan et al., 2008). In summary, *Angelica sinensis* is a potential natural resource in the treatment of CRC, and the question of whether it possesses synergistic effect with OXA needs to be investigated.

Supercritical fluid extract (SFE) is a common method to gather non-polar substances from plants, especially for heat-sensitive compounds. According to previous research, more active ingredients (e.g., Z-ligustilide and 3-butylidenephthalide) can be obtained in *Angelica sinensis* by SFE than other conventional extraction methods (Kim et al., 2006; Saw et al., 2013). Therefore, this study will primarily extract *Angelica sinensis* using SFE, namely, A-SFE, for subsequent experiments.

Cross-communication between cancer cells and macrophages within the tumor microenvironment (TME) plays a critical role in the progression of cancers, including CRC (Yang and Xing, 2021). Under normal circumstances, monocytes differentiate into M1-type pro-inflammatory macrophages, which play an antitumor role. However, monocytes in the TME are (Colvin et al., 2022) influenced by tumor-derived cytokines (i.e., M-CSF, IL-10, IL-6, and TGF-β) and differentiate into tumor-promoting M2-type macrophages (i.e., tumor-associated macrophages, TAMs; Quail and Joyce, 2013). Thus, to mimic the complex interrelationship among macrophages and cancer cells in the TME, we built *in vitro* co-culture systems of HCT116 CRC cells and M2-type macrophages derived from the human monocyte leukemia cell line THP-1. This co-culture system could relatively reflect the anti-cancer and anti-metastasis effects of drugs in TME. Because inhibiting macrophage transformation toward the M2 phenotype could be an efficient therapeutic intervention for cancer (Zhao et al., 2020), flow cytometry was applied to investigate whether A-SFE could inhibit the differentiation of THP-1 into M2-type macrophages, and thus play an anti-cancer role.

This project aims to explore the feasibility of the combination of A-SFE and OXA in the treatment of CRC. We will first focus on the direct anti-cancer effect of A-SFE, and the synergistic effect of SFE and OXA *in vitro*. Their combined efficacy on CT26-induced syngeneic murine tumor will then be investigated. The preliminary mechanism involving TAMs in TME will then be explored based on the syngeneic model.

## Materials and methods

### Reagents and materials

Z-ligustilide was purchased from Shanghai Yuanye Biotechnology Co., LTD., purity ≥98%, No. B20492. Oxaliplatin was purchased from Jiangsu Hengrui Pharmaceutical Co., LTD. (Lot: 190502AM, Jiangsu, China). FBS, RPMI 1640 and high glucose DMEM were purchased from Biological Industries, Israel. Phorbol ester (PMA, phorbol-12-myristate-13-acetate) was purchased from Solarbio (No. P6741, Beijing, China). IL-4 was purchased from PeproTech (No. 200-04-5, New Jersey, USA). Matrigel^®^ matrix was purchased from Discovery Labware (No. 356234, New Jersey, USA). PE-labeled anti-CD206 antibody was purchased from BioLegend (No. 321106, San Diego, USA). Mouse anti-Ki67 (No.ab16667) and CD206 (No.ab64693) were purchased from Abcam (Cambridge, United Kingdom). Mouse anti-MMP9 were purchased from Wuhan Sanying Biology Technology Co., Ltd. (No.10375-2-AP, Wuhan, China).

The HCT116 cell line and THP-1 cell line were obtained from the Cell Resource Center, Chinese Academy of Medical Sciences (Beijing, China). The CT26 cell line was obtained from the Ningbo Mingzhou Biotechnology Co., Ltd. (Ningbo, China).

### Preparation and GC/MS analysis of A-SFE

*Angelica sinensis* was purchased from Beijing Sanhe Yaoye Co., Ltd. (Beijing, China), which was produced in Min County, Gansu Province. They were identified at the Beijing University of Chinese Medicine as per the identification standard of the Pharmacopoeia of the People’s Republic of China, 2020. *Angelica sinensis* was ground into 40 mesh particles. The extraction was conducted using a HA220-50-06 supercritical fluid extraction system (Hua’an Supercritical Extraction Co., Ltd., Nantong, China). When the temperatures in both the extraction and the separation vessels met the requirement, liquid CO_2_ was pumped into the extraction system at a flow rate of 25 L/h. The pressure and temperature of the first separation vessel were 8 MPa and 55°C. The pressure and temperature of the second separation vessel were system tail pressure and 35°C, respectively. After extraction, the products A-SFE were collected from the first separation vessel, weighed, and stored at −20°C for further analysis.

GC/MS analysis of A-SFE was carried out on Agilent 7890B GC system coupled with 5977A mass selective detector (Agilent Technologies Inc., Santa Clara, CA, USA) in the electronic ionization mode (ionization energy: 70 eV). The GC column was an Agilent 19091S-433UI (30 m × 0.25 mm, 0.25 μm). A-SFE was dissolved in ethyl acetate for analysis. The heating temperature was as follows: raise 80°C–167°C at a rate of 3°C/min and hold for 2.5 min, then raise to 202°C at a rate of 2°C/min and hold for 3 min, and finally raise to 280°C at a rate of 4°C/min and hold for 15 min. The inlet temperature and transmission line temperature were both 250°C. Helium was used as carrier gas at a flow rate of 1 ml/min. The injection volume was 1 μL with a split ratio of 10:1. Ion source temperature was 230°C and quadrupole the temperature was at 150°C. The scan scale was 30–600 amu.

### Cell culture and treatment

HCT116 cells and THP-1 cells were cultured in RPMI 1640 and CT26 cells were cultured in high glucose (25 mM) DMEM at 37°C, 5% CO_2_. Both RPMI 1640 and high glucose DMEM were supplemented with 10% FBS and 1% penicillin/streptomycin to prepare the respective complete medium.

### Cell viability assay

The MTT cell viability assay was conducted first to screen the concentrations without cytotoxicity based on Mosmann’s method with minor modifications (Mosmann, 1983). HCT116 cells were seeded in 96-well plates (5 × 10^3^ cells/well) and cultured for 24 h. Then, cells were incubated with gradient concentrations of A-SFE or Z-ligustilide for 24 h. The optical density was measured at 575 nm using a microplate reader system (SPECTROstar Nano, BMG LABTECH, Ortenberg, Germany). The medium group contained only complete medium and the vehicle group contained 0.5% DMSO in complete medium. The cell viability was calculated as follows:

$$Viability\%=\left(OD-{OD}_{0}\right)\;/\;\left({OD}_{c}-{OD}_{0}\right)\times 100\%,$$ (1)

where OD represents the average optical density of samples at the same concentration, OD_0_ represents the optical density of the 96-well plate, and OD_c_ represents for the average optical density of vehicle controls (0.5% DMSO in medium).

A-SFE was dissolved in DMSO to form a 51.2 mg/mL stock solution. The concentrations of A-SFE in cell viability assay were at a range of 2–256 μg/ml, and those of Z-ligustilide ranged from 1.52 to 194.80 μg/ml. The noncytotoxic concentration of A-SFE and Z-ligustilide were chosen for subsequent experiments.

Before investigating the combination effect of OXA and A-SFE, concentrations of OXA ranging from 1 to 128 μg/ml were applied in cell viability assay at first to screen the concentrations, resulting in an inhibition rate of no more than 80%. These selected concentrations were further applied together with A-SFE to evaluate the combined effect. CompuSyn software (ComboSyn, Inc.) was used to calculate the combination index (CI) of A-SFE and OXA according to the cell viability measured by single use and combined use. A CI value of less than 1 indicates synergism, 1 < CI < 1.1 indicates an additive effect, and CI > 1.1 indicates antagonism (Raimundo et al., 2018).

### Cell scratch assay

A cell scratch test was performed to observe the cell’s migration ability. The cells were added to six-well plates. After 24 h cultivation, a ruler was used to scratch the line with the pipette tip (Wang and Li, 2021). The cells were washed three times with PBS to remove the underlined cells and medium supplied with 2% FBS was added. The cells were then incubated in 37°C incubator with 5% CO_2_. Samples were observed and photographed at 24 h. The ImageJ software (National Institutes of Health) was used to analyze the pictures, and the cell migration rate was calculated according to the area of the scratch (S) in the image according to Formula 2:

$$Relative\;cell\;migration\;rate=\left(S_{0h-drug}–S_{24h-drug}\right)\;/\;\left(S_{0h-blank}–S_{24h-blank}\right)\times 100\%.$$ (2)

### Invasion evaluation

Transwell assay was used to evaluate whether A-SFE could inhibit the migration of HCT116 cells. In total, 1 × 10^5^ cells/well were seeded in 200 μL of FBS-free medium precoated with Matrigel in the upper chamber. The lower well was filled with 1 ml of completed DMEM to attract cell invasion. After incubation for 24 h, the cells on the surface of the membrane were fixed with 4% paraformaldehyde, stained with crystal violet, and observed under a microscope. A total of five random high-power microscopic fields were taken for each filter and analyzed by ImageJ. The size (inch^2) was set to 70 infinity in the Analyze Particles in ImageJ to calculate the number of cells.

### M2-polarized THP-1 macrophages

To generate M2-polarized THP-1 macrophages, THP-1 cells were initially cultured with PMA for 48 h and then treated with IL-4 for 72 h (Tjiu et al., 2009). To investigate the effect of A-SFE on inhibiting macrophage to polarize to M2 type, A-SFE was added with IL-4 at the same time in A-SFE group. The cells were then incubated with PE-labeled anti-CD206 antibody to determine the expression of CD206 by flow cytometry (BD FACSCantoⅡFlow Cytometer, USA).

### Co-culture assay

The co-culture of M2 macrophages and HCT116 cells was performed by a Transwell co-culture system. Briefly, HCT116 cells and M2 macrophages were cultured in the lower chamber and upper chamber, respectively. The ratio of M2 cells to HCT116 was 1:1. After co-culture for 48 h, the scratch and invasion experiments were carried out.

### *In vivo* animal experiment

All of the animal experiments were performed in accordance with the NIH Guide for the Care and Use of Laboratory Animals and were approved by the Ethics Committee of Beijing University of Chinese Medicine (Ethical Approval Number: BUCM-4-202112202-4158).

The experiments were performed under a relative humidity rh = (50 ± 10)% and an ambient temperature (25 ± 2)°C with 12-h light/12-h dark in SPF environment. After one week of adaptive feeding, 30 8-week-old male Balb/C mice were divided into five groups, each group consisting of six mice. Then, 1 × 10^6^ CT26 cells/mouse was injected into the right armpit of mice to build a syngeneic tumor model.

The gavage dosage of *Angelica sinensis* was used based on the recommended dosage for humans (12 g/day) according to the Chinese Pharmacopeia 2020, multiplied by the mouse/human body mass ratio equal to 2 g/kg. According to the extraction rate of SFE (1.81%), the low dose of A-SFE (A-SFEL) was determined as 36.2 mg/kg, and the high dose of A-SFE (A-SFEH) was double the low dose at 72.4 mg/kg. Samples were administered by oral gavage once daily. OXA were administered by intraperitoneal injection at a dose of 5 mg/kg every 3 days. Both OXA and A-SFE administration began the day after CT26 cells were injected. The animal grouping and dosing regimen are summarized in Table 1. The weights of each mouse in each group were measured every 2 days. The long diameter 1) and short diameter 2) of the tumors were measured with a Vernier caliper. The tumor volume was calculated based on Formula 3. At the termination of the experiment, the tumors were excised and weighed to record tumor weight. The tumor inhibition rate was calculated based on tumors weight (m) (Formula 4).

$$Tumor\;volume={ab}^{2}/2$$ (3)

$$Tumor\;inhibition\;rate=\left(1-m_{drug\;group}/m_{control\;group}\right)\times 100\%$$ (4)

**TABLE 1:** Animal grouping and dosing regimen (*n* = 6).

Group	Regimen	
	Model (day 0)	Treatment (start from day 1)
Blank	PBS	-
Control	1 × 10^6^ CT26 cells/mouse	-
OXA	1 × 10^6^ CT26 cells/mouse	Oxaliplatin (5 mg/kg every 3 days)
OXA + ASFEL	1 × 10^6^ CT26 cells/mouse	Oxaliplatin (5 mg/kg every 3 days) + A-SFE (36.2 mg/kg daily)
OXA + ASFEH	1 × 10^6^ CT26 cells/mouse	Oxaliplatin (5 mg/kg every 3 days) + A-SFE (72.4 mg/kg daily)

The tumors were embedded in paraffin and processed for HE staining as described. Briefly, tissue sections on glass slides were rehydrated with xylene and alcohol, and counterstained with hematoxylin and eosin to label nuclear and cytoplasm, respectively. Tissue samples were observed under a microscope (Liu et al., 2021).

Immunohistochemistry was performed as previously described (Eom et al., 2011). The primary antibodies were mouse anti-Ki67, mouse anti-CD206, and mouse anti-MMP9.

### Statistical analysis

Statistical significance was calculated using GraphPad Prism (GraphPad Software Company). Mean values were compared using one-way analysis of variance (ANOVA) and unpaired Student’s t-test. *p* < 0.05 was considered to indicate statistical significance. The data were expressed as mean ± SD, unless otherwise specified.

## Results

### Identification of major components of A-SFE

A-SFE from *Angelica sinensis* was obtained by supercritical fluid extraction with yield of 1.81% (v/w) as a pale-yellow viscous oil with pungent odor. The qualitative analysis of A-SFE was based on NIST14 Standard Reference Database and PubChem online databases. As shown in Table 2, 32 compounds in A-SFE were identified, accounting for more than 90% of the total peak area. The component with the largest normalized peak area was Z-ligustilide (70.23%), followed by E-ligustilide (10.89%).

**TABLE 2:** Qualitative analysis of *Angelica sinensis* supercritical fluidextract (A-SFE) based on GC/MS analysis.

No.	Retention time (min)	Compound name	Molecular formula
1	17.069	Tridecane	C_13_H_28_
2	18.151	Elemene	C_15_H_24_
3	18.698	Campholenic aldehyde	C_10_H_17_NO
4	20.198	γ-Elemene	C_15_H_24_
5	20.704	Trans-Sesquisabinene hydrate	C_15_H_26_O
6	23.309	(-)-Spathulenol	C_15_H_24_O
7	25.533	(+)-Spathulenol	C_15_H_24_O
8	26.062	Butylphthalide	C_12_H_14_O_2_
9	26.339	1,3-Cyclohexadiene-1-methanol, α,2,6,6-tetramethyl	C_11_H_16_O
10	26.791	1(3H)-Isobenzofuranone, 3- butylidene	C_12_H_16_O_4_
11	28.462	Senkyunolide	C_12_H_16_O_2_
12	29.097	Z-ligustilide	C_12_H_14_O_2_
13	31.326	E-ligustilide	C_12_H_14_O_2_
14	32.85	Dipentadecyl ketone	C_31_H_62_O
15	39.026	n-Hexadecanoic acid	C_16_H_32_O_2_
16	39.667	Benzenepropionic acid, 4-tridecyl ester	C_22_H_36_O_2_
17	44.326	Linoleoyl chloride	C_18_H_31_ClO
18	46.384	Linoleic acid	C_18_H_32_O_2_
19	47.225	Ethyl linoleate	C_20_H_36_O_2_
20	49.649	Panaxydol	C_17_H_24_O_2_
21	52.131	2,2-Dimethyl-6-methylene-1- [3,5-dihydroxy-1-pentenyl]cyclohexan-1-perhydrol	C_14_H_24_O_4_
22	52.36	2(5H)-Furanone, 4-methyl3,5,5-tris(2-methyl-2-propenyl)-	C_17_H_24_O_2_
23	59.801	Cholest-5-en-3-ol (3β)-, tetradecanoate	C_41_H_72_O_2_
24	61.16	4,7,7-Trimethyl-3-oxo-2- oxabicyclo[2.2.1]heptane-1-carboxylic acid, 2,4,4- trimethyl-3-(3-oxo-but-1- enyl)-cyclohex-2-enyl ester	C_23_H_32_O_5_
27	62.748	13-Octadecenal	C_18_H_34_O
28	65.389	2H-3,9a-Methano-1-benzoxepin, octahydro-2,2,5α,9- tetramethyl-, [3R- (3α,5α,9α,9α)]-	C_15_H_26_O
29	65.912	Abscisic acid	C_15_H_20_O_4_
30	67.783	Trans-9-octadecenoic acid, pentylester	C_23_H_44_O_2_
31	70.142	9-Octadecenoic acid (Z)-, 2-hydroxy-1-(hydroxymethyl)ethylester	C_21_H_40_O_4_
32	74.73	10-Octadecenoic acid, methylester	C_20_H_38_O_2_

**FIGURE 1:**
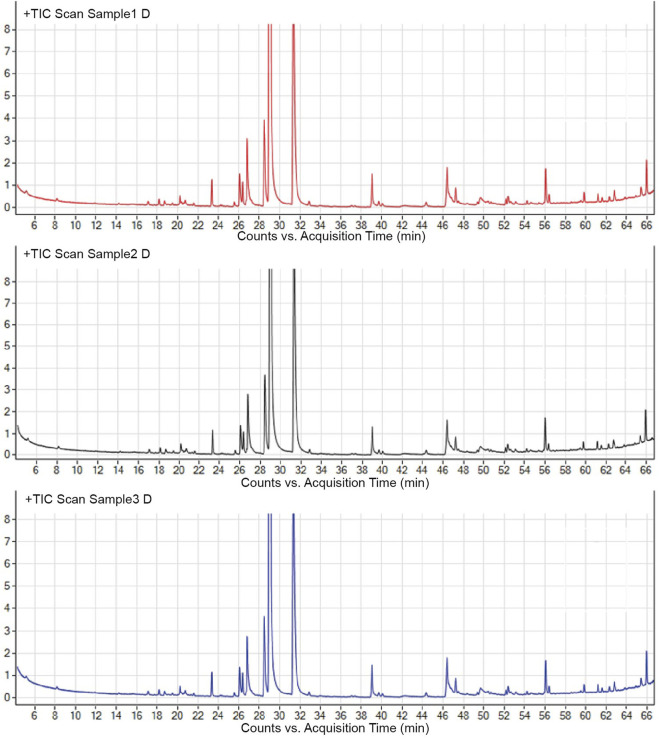
Major components in A-SFE. GC/MS total ion chromatogram of three batches of A-SFE.

To validate the stability of the process, the A-SFE was extracted three times and analyzed by GC/MS (Figure 1). The normalized area of each peak was stable with relative standard deviation (RSD) less than 10% by GC/MS analysis. Hence, A-SFE with stable quality was acquired, which could be further studied.

### Direct effect of A-SFE and its main compounds on HCT116 *in vitro*

**FIGURE 2:**
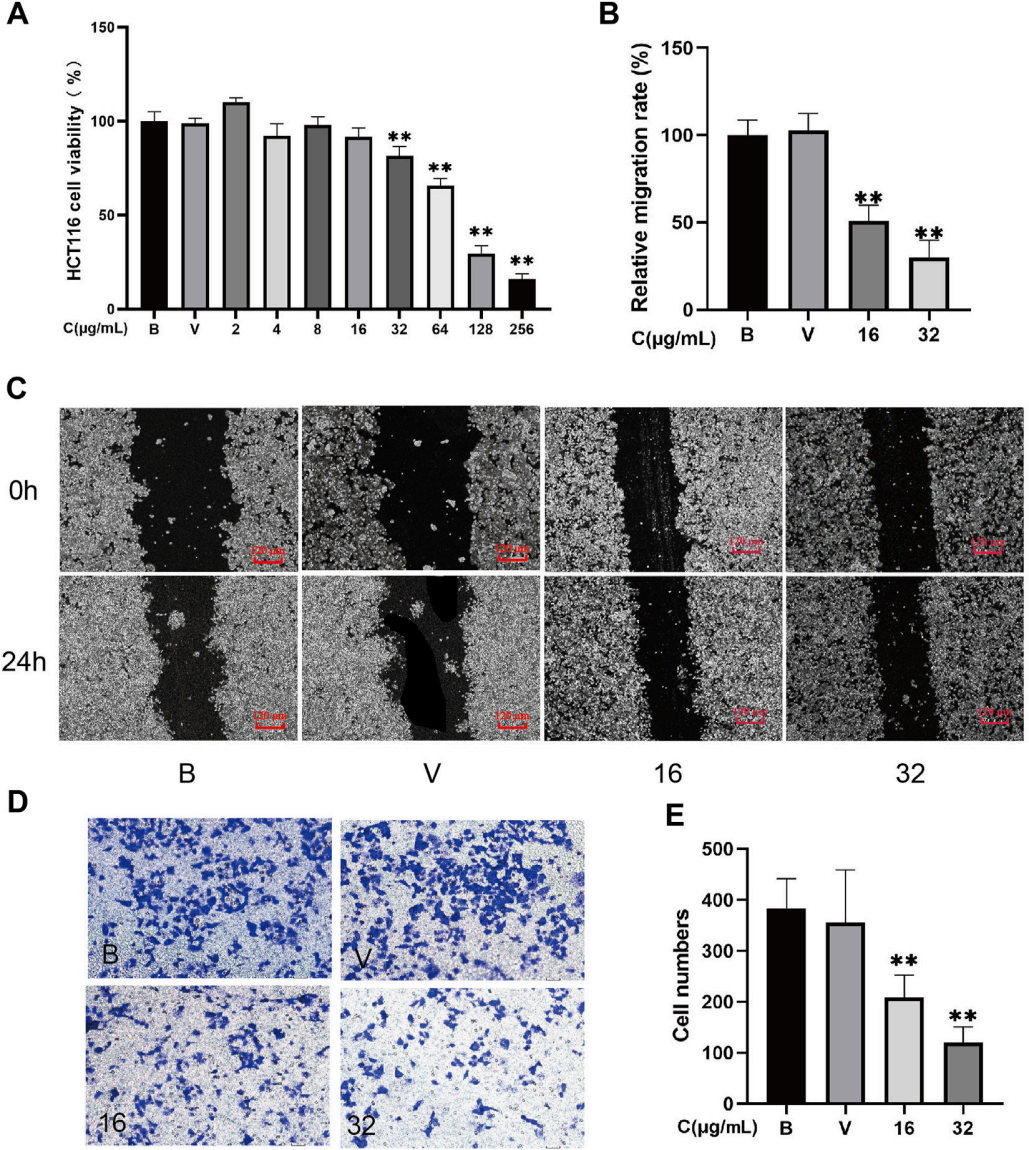
A-SFE directly inhibited the proliferation, metastasis, and invasion of HCT116 cells (*n* = 5). **(A)** Cell viability of HCT116 at different concentrations of A-SFE in MTT assay. **(B)** Relative migration rate calculated using Formula 2. **(C)** Representative image of the *in vitro* scratch migration assay of HCT116 cells after 0 or 24 h evaluated by phase microscopy. **(D)** Image of cells stained with crystal violet in Transwell invasion assay. **(E)** Number of cells that successfully crossed Matrigel and invaded into the lower chamber in each group. (B: blank group, untreated; V: vehicle group, treated with 0.5% DMSO in medium; 16: treated with 16 μg/mL A-SFE; 32: treated with 32 μg/mL A-SFE). **p < 0.01 indicates significant differences compared with the vehicle group.

MTT analysis demonstrated that 32–256 μg/mL A-SFE dose-dependently inhibited the viability of HCT116 cells (*p* < 0.01). After 24 h of incubation, the IC_50_ value for A-SFE in HCT116 cells was 84.51 μg/ml. Cell viabilities of HCT116 were greater than 80% under 0–32 μg/mL A-SFE (Figure 2A). To investigate the effect of A-SFE on migration, we performed the scratch wound-healing assay.

To exclude the drug’s toxicity interfering in migration and invasion assay by inhibiting cell proliferation, 16 μg/mL and 32 μg/mL A-SFE that caused greater than 80% cell viabilities of HCT116 were chosen for subsequent experiments. To investigate the effect of A-SFE on migration, we performed the scratch wound-healing assay (Figures 2B,C). The scratch assay showed that scratches in HCT116 cells were partially healed after 24 h. The area of the scratch was analyzed using ImageJ software. The results show that when compared with blank HCT116 (100%), the healing capacity was decreased in HCT116 cells that were treated by 16 μg/ml or 32 μg/mL A-SFE ((50.86 ± 8.06)% and (30.04 ± 8.77)%, *p* < 0.01). These results suggested that 16 μg/ml or 32 μg/mL A-SFE inhibited the migratory ability of HCT116 cells. Moreover, treatment with A-SFE significantly suppressed the invasion of HCT116 cells across Matrigel (*p* < 0.01 compared to vehicle treatment; Figures 2D,E).

**FIGURE 3:**
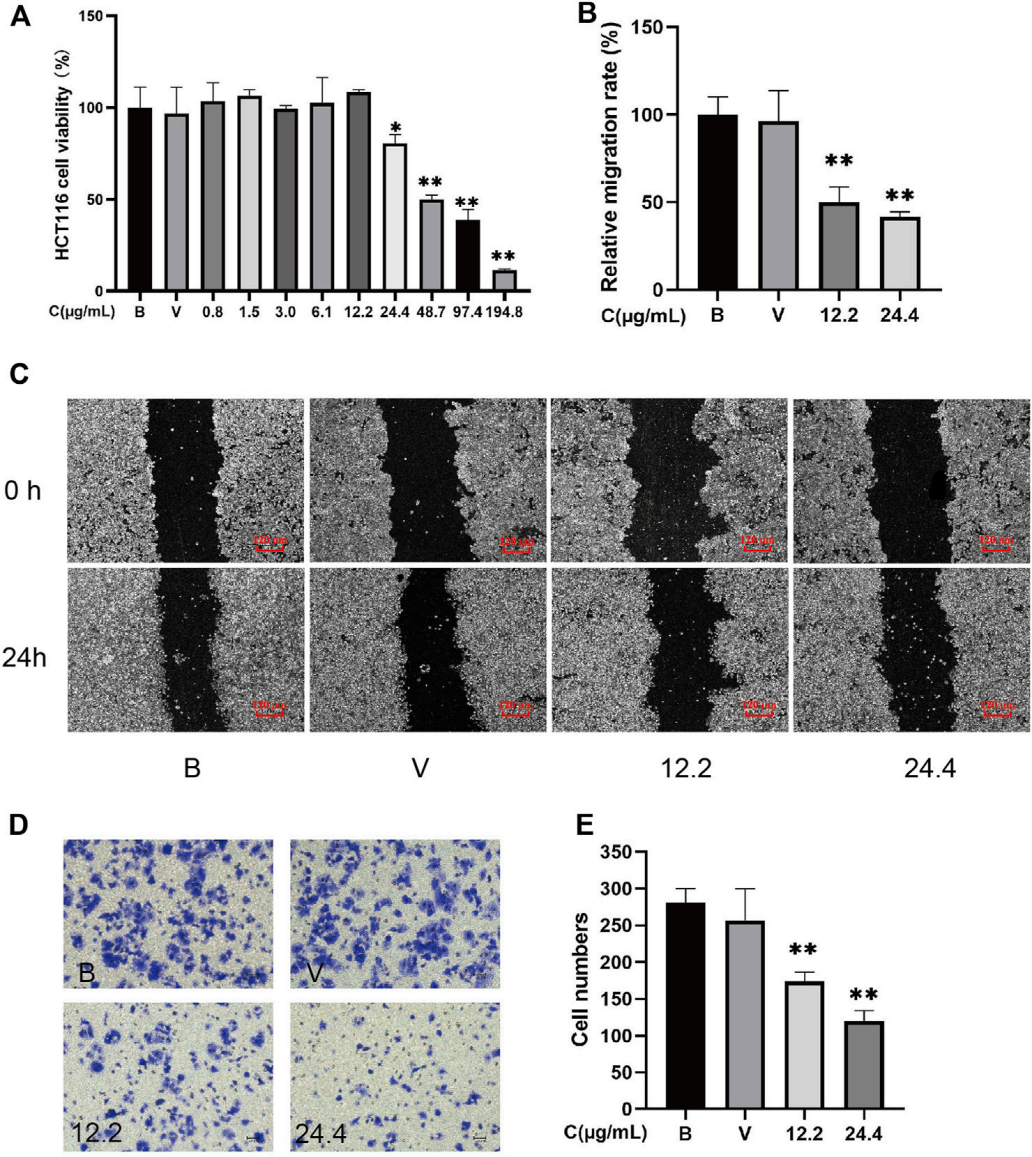
Z-ligustilide directly inhibited the proliferation, metastasis, and invasion of HCT116 cells (*n* = 5). **(A)** Cell viability of HCT116 at different concentrations of Z-ligustilide in MTT assay. **(B)** Relative migration rate calculated using Formula 2. **(C)** Representative image of the *in vitro* scratch migration assay of HCT116 cells after 0 or 24 h evaluated by phase microscopy. **(D)** Image of cells stained with crystal violet in the Transwell invasion assay. **(E)** Number of cells that successfully crossed Matrigel and invaded into the lower chamber in each group. (B: blank group, untreated; V: vehicle group, treated with 0.5% DMSO in medium; 12.2: treated with 12.2 μg/ml Z-ligustilide, 24.4: treated with 24.4 μg/ml Z-ligustilide). *p < 0.05, **p < 0.01 indicate significant differences compared with vehicle group.

MTT analysis demonstrates that 24.4–194.8 μg/ml Z-ligustilide dose-dependently inhibited the viability of HCT116 cells (*p* < 0.01). After 24 h of incubation, the IC_50_ value for Z-ligustilide in HCT116 cells was 60.25 μg/ml. The cell viability of HCT116 is greater than 80% under 0–24.4 μg/ml Z-ligustilide (Figure 3A). To investigate the effect of Z-ligustilide on migration, we performed a scratch wound-healing assay.

To exclude the drug’s toxicity interfering in migration and invasion assay by inhibiting cell proliferation, 12.2 μg/ml and 24.4 μg/ml Z-ligustilide that caused greater than 80% cell viabilities of HCT116 were chosen for subsequent experiments. To investigate the effect of Z-ligustilide on migration, we performed a scratch wound-healing assay (Figures 3B,C). The scratch assay showed that scratches in HCT116 cells were partial healed after 24 h. The results from ImageJ software analysis show that when compared with blank HCT116 (100%), the healing capacity was decreased in HCT116 cells that were treated by 12.2 μg/ml or 24.4 μg/ml Z-ligustilide ((50.06 ± 8.62)% and (41.85 ± 2.66)%, *p* < 0.01). These results suggested that 12.2 μg/ml or 24.4 μg/ml Z-ligustilide inhibited the migratory ability of HCT116 cells. Moreover, treatment with Z-ligustilide significantly suppressed the invasion of HCT116 cells across Matrigel (*p* < 0.01 compared to blank or vehicle treatment; Figures 3D,E).

### Alteration on M2-type polarization of macrophages by A-SFE *in vitro*

**FIGURE 4:**
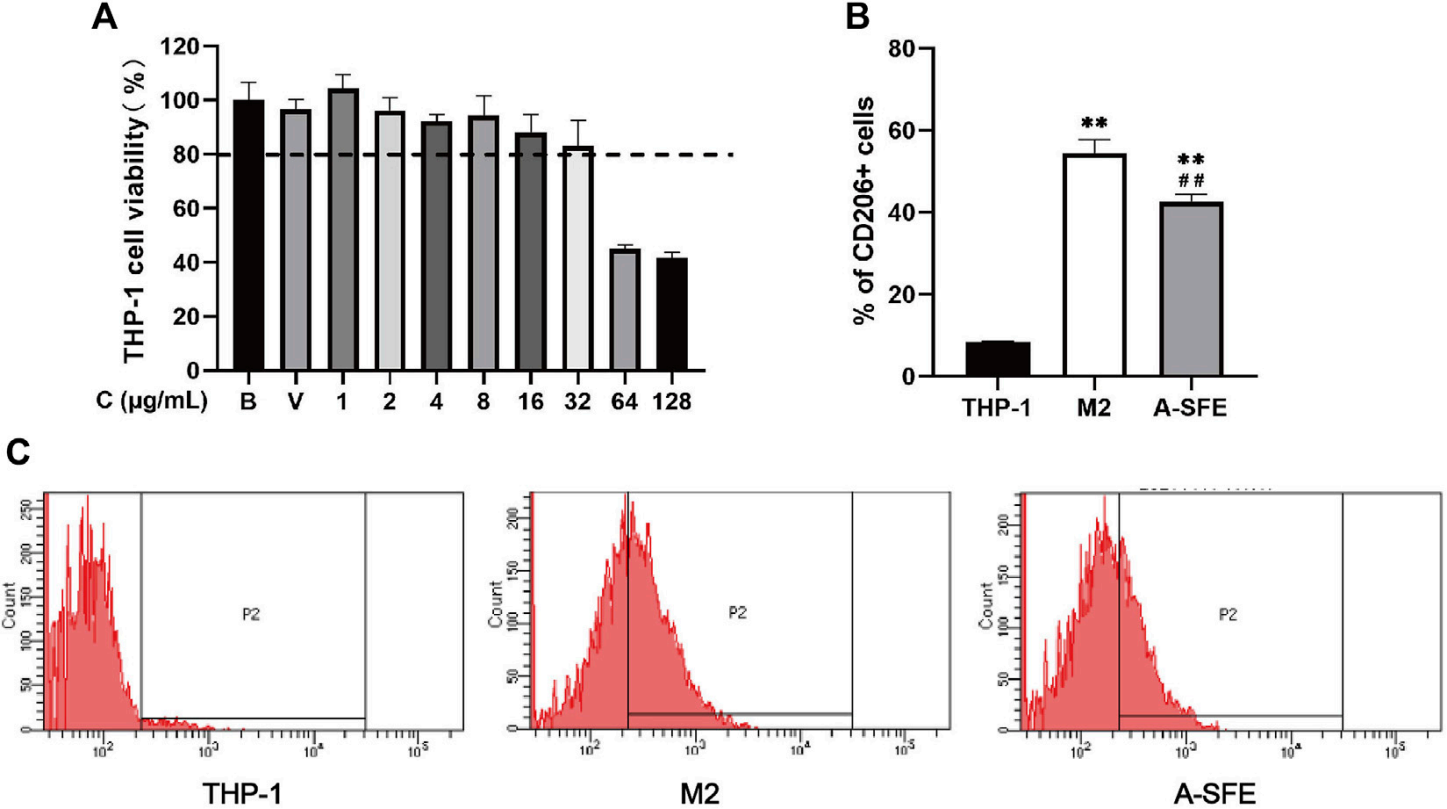
A-SFE inhibited M2 polarization in THP-1 monocyte-derived macrophages (*n* = 3). **(A)** Cell viability of THP-1 at different concentrations of A-SFE in MTT assay. **(B)** Proportion of CD206-positive cells in flow cytometry. **p < 0.01 indicates significant differences compared with THP-1 group. ##*p* < 0.01 indicates significant differences compared with the M2 group. **(C)** Histograms plot of each group’s through flow cytometry. THP-1 group (no treated). M2 group (THP-1 was treated by PMA and IL-4 to induce to M2 macrophages). A-SFE group (administered by 32 μg/mL A-SFE on the basis of the M2 group).

Before the polarization experiment, the toxicity of A-SFE to THP-1 was determined and the concentrations of cell viability greater than 80% were selected for subsequent experiments (Figure 4A).

M2-type macrophages account for the majority of TAMs. Thus, inhibiting M2-type cells is considered to be an effective therapeutic strategy in cancer therapy. We incubated HCT116 with PMA for 48 h, followed by IL-4 for 72 h to polarize the cells to an M2-type phenotype. To investigate the effect of A-SFE on inhibiting macrophage polarize to M2, A-SFE (32 μg/ml) was added with IL-4 at the same time in the A-SFE group. Polarization corroboration by flow cytometric analysis was performed to examine the levels of polarization-related surface markers CD206^high^, which are characteristic of M2 macrophages. Treatment with A-SFE resulted in significantly reduced levels of CD206 in M2-type macrophages (Figures 4B,C) (Table 3).

**TABLE 3:** Percentages of CD206+ M2-like macrophages in each group ( $\bar{x}$ ± *s*, *n* = 3).

Group	CD206+ (%)
THP-1	8.30 ± 0.30
M2	54.50 ± 3.20
A-SFE	42.60 ± 1.80

### Effect of A-SFE on colorectal cancer in cell co-culture system

**FIGURE 5:**
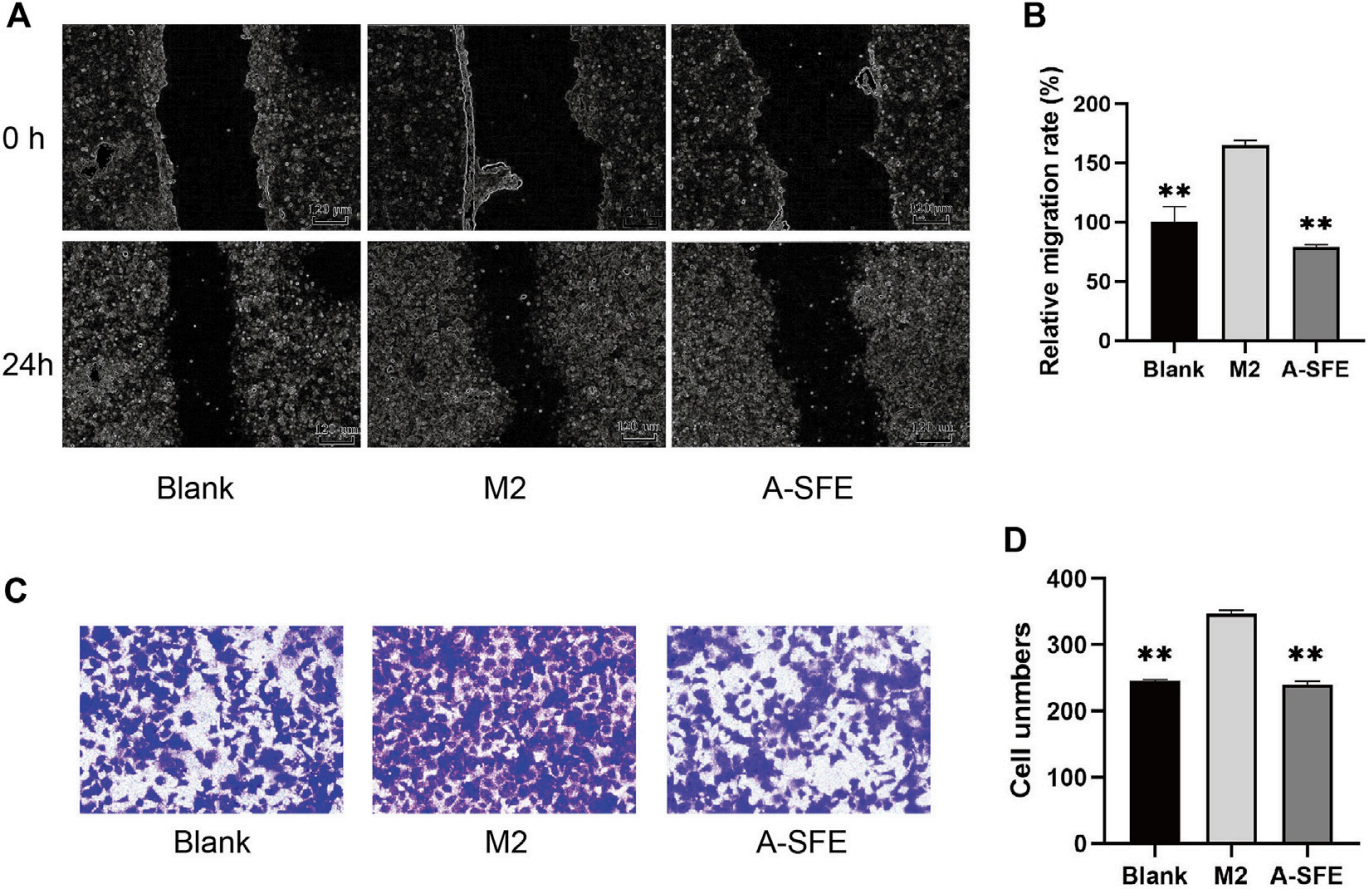
A-SFE inhibited the migration and invasion of HCT116 in the co-culture system with THP-1-derived M2 macrophages (*n* = 3). **(A)** Representative images of the *in vitro* scratch migration assay in HCT116 cells immediately after the wounding (0 h), HCT116 cells only (blank group) or co-culture with M2 macrophages induced from THP-1 (M2 group), added A-SFE in the co-culture with M2 macrophages (A-SFE group) after 24 h incubation evaluated by phase microscopy. **(B)** Relative migration rate of the scratch assay calculated using Formula 2. **(C)** Image of HCT116 cells in co-culture model stained with crystal violet in the Transwell invasion assay. **(D)** Number of cells that successfully crossed Matrigel and invaded into the lower chamber in each groups. **p < 0.01 indicates significant differences compared with the M2 group.

On the basis of our results, we suggest that A-SFE could not only suppress proliferation and migration of HCT116 cells but could also inhibit M2 polarization of THP-1. To simulate cross-communication between cancer cells and macrophages within TME, and reflect the anti-cancer and anti-metastasis effect of drugs in the TME, we built a Transwell co-culture system of HCT116 and macrophages M2 from THP-1. M2-polarized THP-1 macrophages were cultured in the upper chamber and HCT116 were cultured in the lower chamber. Then, *in vitro* scratch assay was performed in the lower chamber (Figures 5A,B). HCT116 cells in the co-culture with M2 macrophages (M2 group) showed stronger migration capabilities on scratch assay than when cultured alone (blank group). A-SFE inhibited HCT116 scratch wound healing in the co-culture with M2 macrophages (A-SFE group). In the Transwell invasion assay, M2-polarized THP-1 macrophages were cultured in the lower chamber and HCT116 were cultured in the upper chamber. We then observed the number of HCT116 cells that invaded Matrigel and reached the lower chamber (Figures 5C,D). The results show that the number of HCT116 cells that invaded into the lower chamber in the co-culture of M2 macrophages was significantly higher than when cultured alone (group blank). The number of HCT116 cells invading the lower chamber was significantly reduced under M2 macrophage co-culture after administration of A-SFE (group A-SFE). This indicates that A-SFE effectively inhibited the metastasis and invasion ability of HCT116 in the co-culture system.

### Synergistic effect of A-SFE and OXA in combination

**FIGURE 6:**
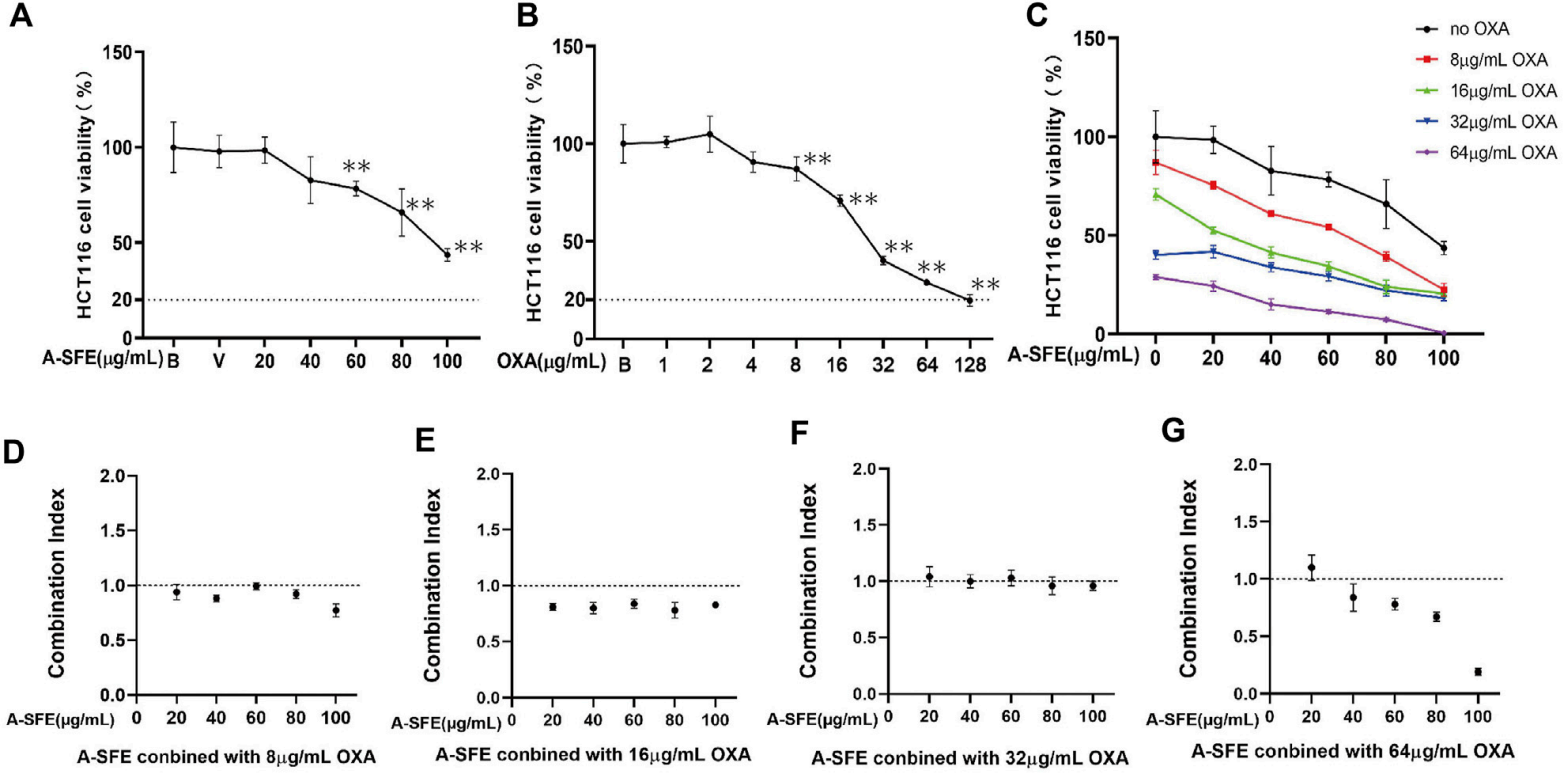
OXA and A-SFE showed synergistic effect and additive effect in HCT116 cells (*n* = 5). Cell viabilities of HCT116 at different concentrations of oxaliplatin **(A)** or A-SFE **(B)** in MTT assay. **p < 0.01 indicates significant differences compared with blank or vehicle. **(C)** Cell viability of HCT116 under the combined effects of 0, 8, 16, 32, 64 μg/ml of OXA and different concentrations of A-SFE. **(D–G)** Combination index of synergy between OXA and A-SFE.

The single inhibitory effect of OXA and A-SFE on HCT116 was first determined using the MTT assay (Figures 6A,B). A-SFE and OXA dose that caused more than 20% cell viabilities of HCT116 were chosen for the combination experiments. Meanwhile, 8, 16, 32, and 64 μg/ml of OXA and 20, 40, 60, 80, and 100 μg/ml of A-SFE were selected to measure the viability of HCT116 cells in the combination experiments (Figure 6C). A significantly greater decrease in cell viability was identified in the combined-agent group when compared with the single-agent groups. CompuSyn software was used to calculate the combination index (CI) of A-SFE and OXA according to the cell viability measured by single use and combined use (Figures 6D–G). A CI value of less than 1 indicates synergism, 1 < CI < 1.1 indicates an additive effect, and CI > 1.1 indicates antagonism (Raimundo et al., 2018). When 8 μg/ml OXA was combined with 20 μg/ml and 60 μg/mL A-SFE, the combination indices were close to 1, showing additive effects. When 8 μg/ml OXA was combined with 40, 80, and 100 μg/mL A-SFE, the combination indices were less than 1, showing synergistic effects (Figure 6D). Meanwhile, 16 μg/ml OXA and 20–100 μg/mL A-SFE showed significant synergy, the combination indices were all less than 0.85 (Figure 6E). The combination of 32 μg/ml OXA and 20–100 μg/mL A-SFE showed additive effects (Figure 6F). When 64 μg/ml OXA was combined with 40–100 μg/mL A-SFE, the combination indices were less than 1, showing synergistic effects (Figure 6G).

### Antitumor efficacy of A-SFE and OXA in combination *in vivo*

**FIGURE 7:**
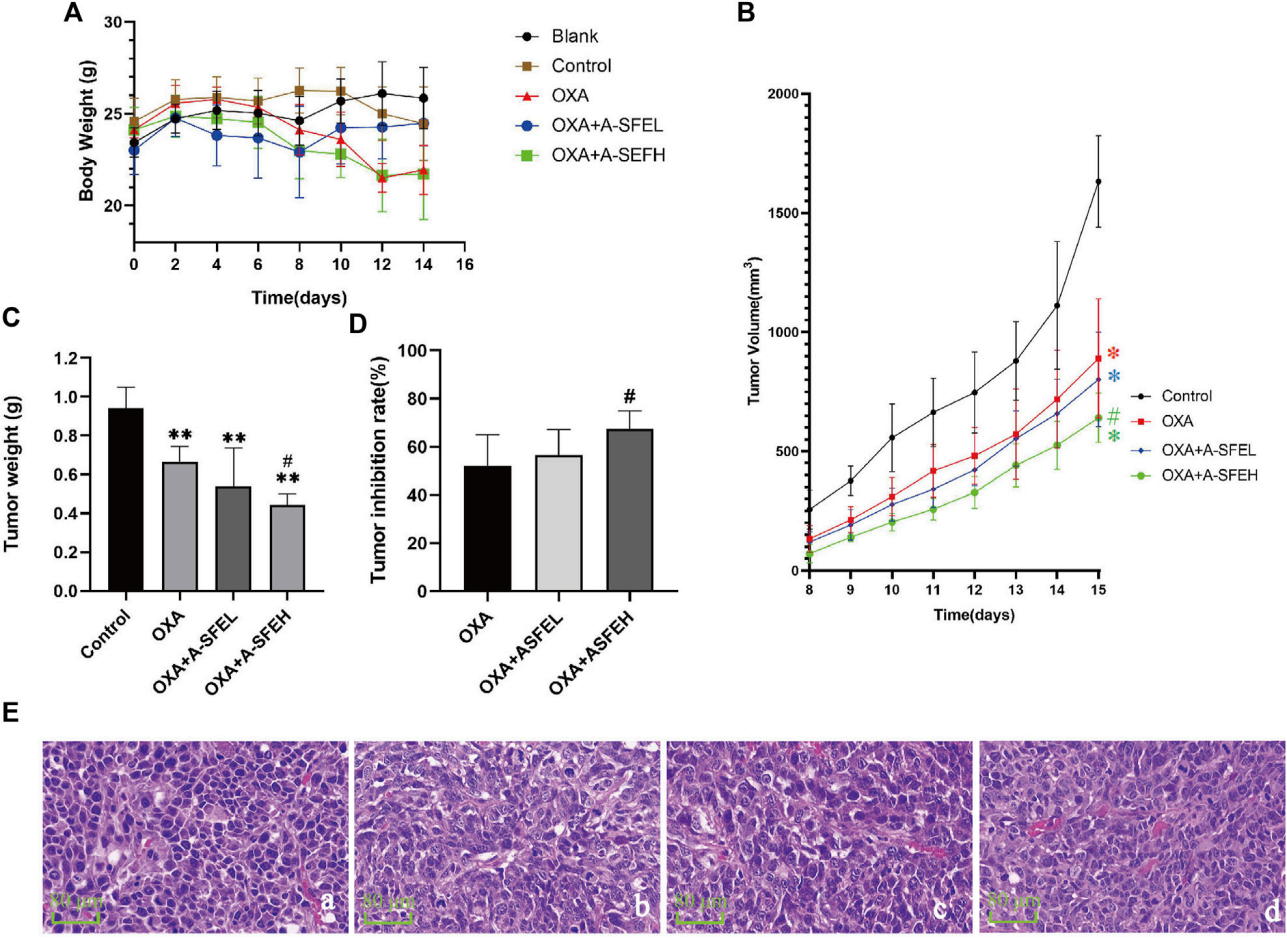
Combination of A-SFE and OXA effectively inhibited tumor growth. **(A)** Line chart of mouse body weight in each group (*n* = 6). **(B,C)** Line chart and histogram of tumor volume (m^3^) in each group (*n* = 6). **(D)** Tumor inhibition rates of tumor volume in each group (*n* = 6). **(E)** Representative images of HE-stained tumor tissue from each group (× 400) (*n* = 2). (a: Control group; b: OXA group; c: A-SFE + ASFEL group; d: OXA + ASFEH group.) **p < 0.01 indicates significant differences compared with control group. #*p* < 0.05 indicates significant differences compared with the OXA group.

The results of the experiment are shown in Figure 7. During the experimental period, the weight of the mice in the blank group gradually increased, while the weight of the other groups increased at first and then decreased (Figure 7A). Starting at day 8, the short and long diameters of the tumors were measured daily and tumor volumes (mm^3^) were calculated. The daily average tumor volume of each group is shown in Supplementary Table S1 and the growth trend of tumor volume in each group is shown in Figure 7B. The average tumor volume of the mice in each group showed an increasing trend, but the growth rate in the control group was significantly faster than that in the drug groups. On day 15, the tumor volume was (1631.50 ± 191.46) mm^3^ in the control group, (889.49 ± 250.516) mm^3^ in the OXA group, (801.60 ± 197.98) mm^3^ in the OXA + A-SFEL group, and (640.69 ± 103.90) mm^3^ in the OXA + A-SFEH group. At the end of the experiment, the tumors were removed and weighed. Images of all of the tumors are shown in Supplementary Figure S1. The tumor weight was (1.3545 ± 0.1398) g in the control group, (0.6489 ± 0.1752) g in the OXA group, (0.5883 ± 0.1427) g in the OXA + A-SFEL group, and (0.4423 ± 0.1016) g in the OXA + A-SFEH group. The average tumor weight in the OXA + ASFEH group was statistically lower than that in the OXA group (Figure 7C). The tumor inhibition rates of the mice in the other administration groups were calculated according to the tumor weight of the mice in the control group (Figure 7D). The tumor inhibition rate of the OXA + A-SFEH group reached 67.35%, which was higher than the OXA group (52.09%). These results indicate that A-SFE could increase the efficacy of OXA against CRC. Representative images of HE-stained tumor tissues are shown in Figure 7E. The HE staining showed that the tumor cells were irregular, deep-colored, and arranged closely with larger and abnormal nuclei and nuclear pleomorphism in the control group. In the OXA and OXA + A-SFE groups, the arrangement of the tumor cells was disordered and there was nuclear pyknosis, necrosis, and dissolution of some cells.

**FIGURE 8:**
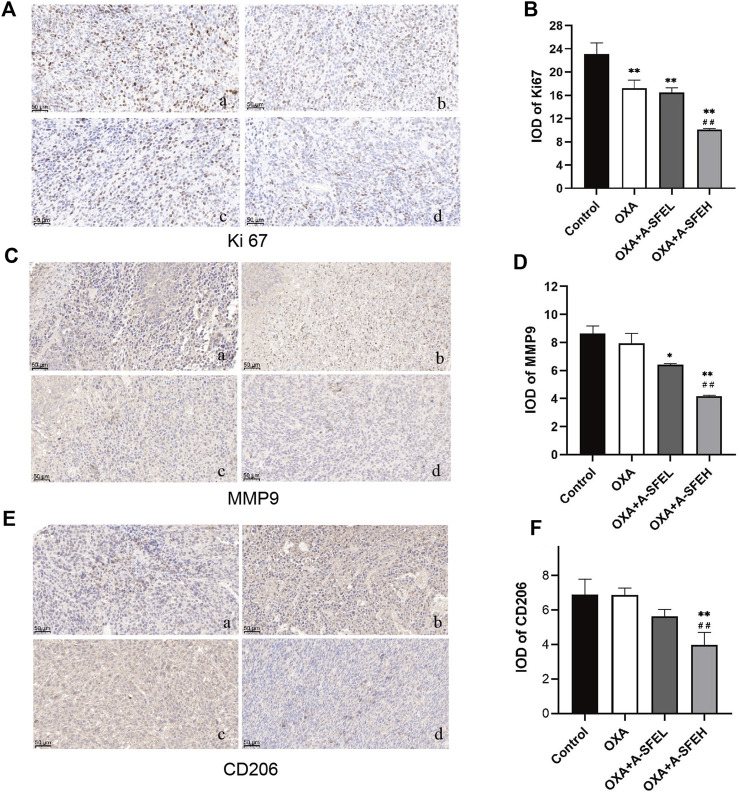
Combination of A-SFE and OXA effectively inhibited the expression of Ki67, MMP9, and CD206 (*n* = 3). Representative pictures of immunohistochemistry staining for Ki67 **(A)**, MMP9 **(C)**, CD206 **(E)** (×200). (a: Control group; b: OXA group; c: A-SFE + ASFEL group; d: OXA + ASFEH group.) **(B,D,F)** Integrated optical density (IOD) analysis of histological sections by Image-Pro Plus 5.0 software. **p < 0.01 and *p < 0.05 indicate significant differences compared with the control group. ##*p* < 0.01 indicates significant differences compared with the OXA group.

We analyzed the expression levels of Ki67, MMP9, and CD206 by immunohistochemistry. Images of five random visual fields from each group were captured under a microscope (×200 magnification). The integrated optical density (IOD) value analysis of positive cells expressed in yellow or yellow-brown in the field of view was carried out using an Image-Pro Plus 5.0 imaging system (Figure 8).

Ki67 was predominantly expressed in the nucleus. Compared with the control group, the expression of Ki67 in tumor-bearing mice in each administration group decreased significantly. This shows that the abnormal expression of Ki67 in tumor tissue could be significantly improved after drug treatment and cell proliferation could be inhibited. The expression of Ki67 in the OXA + A-SFEH group was significantly lower than that in the OXA group, which shows that the combination of OXA and high-dose A-SFE could significantly enhance the efficacy (*p* < 0.01) (Figures 8A,B). MMP9 was mainly expressed in cytoplasm and a few in nuclear. Compared with the control group, using OXA alone had no significant effect on the expression of MMP9. OXA combined with A-SFE significantly reduced the expression of MMP9 protein in a dose-dependent manner (Figures 8C,D). CD206 is a surface marker that is exclusively expressed by M2 macrophages. Compared with the control group, OXA alone had no significant effect on the expression of CD206. The expression of CD206 in tumor tissue of the OXA + ASFEH group was significantly decreased (*p* < 0.01). This indicates that A-SFE could inhibit M2-type polarization of macrophages in tumor tissue in mice (Figures 8E,F).

## Discussion

This article explored the potential of A-SFE as an adjuvant anti-colorectal cancer drug. First, the anti-cancer effects of A-SFE used alone were explored. The experiments show that A-SFE could inhibit the viability of HCT116 cells at 32–256 μg/ml *in vitro*. Scratch assay and Transwell assay confirmed that A-SFE also held certain anti-metastasis and anti-invasion ability. However, the healing ability of HCT116 in the scratch test was not significant and it did not show a significant scratch healing in the images of the scratch migration assay. Considering that immune cells in TME plays an important role in modulating the proliferation, invasiveness, and motility of cancer cells (Neophytou et al., 2021), the viability and metastatic ability of cancer cells could be stronger under the action of tumor-associated immune cells. Therefore, it was necessary to explore the inhibitory effect of A-SFE on HCT116 under the influence of tumor-associated immune cells. Meanwhile, the function of *Angelica sinensis* in regulating immunity has previously been reported (Yang et al., 2006). Ligustilide could change the immunosuppressive function of cancer-associated fibroblasts (CAFs) through the TLR4-NF-κB pathway and restore T-cell proliferation previously inhibited by the CAF supernatant (Ma et al., 2019). It was speculated that A-SFE might exert a more effective anti-cancer effect by regulating immune cells in TME, which was also necessary to explore. TAMs are the main type of myeloid cells implicated in regulating the metastasis of cancer cells in TME (Neophytou et al., 2020). M2-type TAMs are pro-tumorigenic and exert immunosuppressive functions by producing IL-10, induce angiogenesis, and stimulate tumor cells to release MMPs that favor cancer progression by disrupting the ECM and BM (Singh et al., 2017).The inhibitory effect of A-SFE on the polarization of THP-1 to M2 macrophages in the microenvironment and the anti-cancer efficacy of A-SFE in the co-culture environment of M2 and HCT116 cells were investigated *in vitro*. In the flow cytometry experiments, it was found that A-SFE could inhibit the polarization of THP-1 to M2-type macrophages. This confirmed our speculation. The anti-cancer effect of A-SFE was studied in the co-culture system of M2-type macrophages and HCT116. It was found that the migration ability and invasion ability of HCT116 in the co-culture system were stronger than that of HCT116 alone. In contrast, A-SFE significantly inhibited the metastatic and invasive abilities of HCT116 in co-culture. This suggests that A-SFE could regulate TAMs in TME and could also play an anti-metastatic role in TME. This is of great clinical significance for patients with advanced CRC.

In our previous experiments, the anti-HCT116 activity (MTT assay) of Z-ligustilide and A-SFE were compared, and their correspondent IC_50_ values were calculated. Given that Z-ligustilide and A-SFE were extracted from *Angelica sinensis*, IC_50_ was converted into crude drug content for comparison. Crude drug concentration refers to the dosage of crude drug using for extraction (g)/liquid volume (ml). The IC_50_ values of A-SFE and Z-ligustilide in HCT116 cells were 84.51 μg/ml and 60.25 μg/ml. According to the extraction yield and Z-ligustilide content, the IC50 of A-SFE was converted to 4.62 mg crude drug/mL, which was lower than that of Z-ligustilide (11.13 mg crude drug/mL) (Supplementary Table S2). This result indicates that the antitumor effect of A-SFE not only depended on Z-ligustilide but also depended on other components. In addition, the instability of Z-ligustilide limits its further research and application in clinics. As an unsaturated phthalide with a 3-butenyl group, it is prone to isomerize into other similar phthalide isomerized compounds. A stability study on Z-ligustilide showed that after 15 days of storage at room temperature, the purity of Z-ligustilide decreased from 99.98% to 41.97%, and was not detected after another 15 days (Fang et al., 2014). The Z-ligustilide might be more stable in A-SFE, due to the mutual equilibrium of the various phthalide isomers in it (Zhao et al., 2008). In addition, Z-ligustilide is difficult to synthesize and the extraction rate is low, while A-SFE is more reproducible (Zhao et al., 2012). Hence, we chose A-SFE for further study.

Chemotherapy is a very common anti-cancer therapy in clinical practice. In CRC, chemotherapy is often recommended for patients with advanced and metastatic disease. However, the main mechanism of chemotherapy is to directly kill cancer cells, regulate its cell cycle, and promote apoptosis, which has little inhibitory effect on metastasis. Some chemotherapeutic drugs may even promote cancer metastasis (Keklikoglou et al., 2019). In addition to these problems, the most concerning disadvantage of chemotherapeutic drugs such as oxaliplatin is their toxic side effects, especially neurotoxicity, after high-dose use (Jaggi and Singh, 2012). If A-SFE could increase the sensitivity of OXA and synergistically play an anti-cancer effect when used in combination, then it would become an ideal adjuvant anti-cancer drug. The individual and combined inhibitory effects of different concentrations of OXA and A-SFE on HCT116 were investigated by MMT experiments and the synergy coefficient was calculated. The experiments found that the CI of some concentrations of OXA and A-SFE was lower than 0.9, which had a relatively obvious synergistic effect. This study then established a mouse syngeneic model to explore the antitumor effect of OXA combined with A-SFE *in vivo*. The results show that tumor volume and growth rate were significantly lower when A-SFE and OXA were used in combination than when OXA was used alone. This shows that A-SFE can synergize with OXA and thereby the lower OXA dose while still maintaining its efficacy. Finally, to explore the regulatory pathway of A-SFE, this article selected three landmark targets in tumor proliferation, metastasis, and microenvironment to carry out immunohistochemical experiments to explore whether they regulate these key targets. Ki67, as a nuclear marker, is closely associated with tumor cell proliferation and cell division cycle (Booth et al., 2014). MMP9 is increased in tumor foci in the digestive system (Wang and Zhang, 2020; Shang et al., 2021). During the development of cancer, due to its capacity to degrade extracellular protein components, MMP9 is involved in the removal of physical barriers to cell migration. The disruption of the basement membrane (BM), which consists of a dense network of crosslinked laminins and collagens, not only facilitates the spread of invading cancer cells or the influx of immune cells into the tumor stroma but also facilitates the formation of new blood and lymphatic vessels that are necessary for supplying tumor tissue with nutrients and oxygen (Augoff et al., 2022). TAMs play a crucial role in the development of malignancies, which makes it an appealing target for cancer therapy. Macrophages are derived from monocytes. In response to cytokines and other stimuli of their microenvironment, macrophages polarize toward either pro-inflammatory type 1 (M1) or anti-inflammatory type 2 (M2) macrophages. M2 macrophages can inhibit T-cell activation, promote the growth of tumor cells, and are involved in metastasis to distal tissues (Zhao et al., 2020). Thus, inhibition macrophage transformation toward the M2 phenotype could be an efficient therapeutic intervention for cancer. CD206 is a mannose receptor that is expressed by all M2 macrophages (Monteiro et al., 2018). Therefore, this article selected these three key targets and explored the expression of these targets using *in vivo* experiments. The experiments confirm that OXA can effectively inhibit proliferation-related Ki67 but not MMP9 and CD206. The combination of A-SFE and OXA inhibited the expression of Ki67, MMP9, and CD206. The combination of the two drugs was effective in anti-proliferation, anti-metastasis, and modulating the tumor microenvironment, which is consistent with the *in vitro* results.

Currently, chemotherapy methods are mainly recommended to advanced and metastatic patients in the clinical treatment of CRC. There are, however, many problems in the chemotherapy of these patients, including the potential adverse and side effects, as well as the limited effectiveness against metastases (Lehky et al., 2004; McKeage, 1995). The ideal adjuvant drug for cancer patients during chemotherapy is able to play a role in the improvement of these points. The A-SFE studied in this article could synergistically enhance the anti-CRC effect of OXA, both *in vitro* and *in vivo*. The combination of A-SFE with a lower dose OXA may achieve the same efficacy, thereby reducing oxaliplatin-induced adverse events. In the mechanism study, it was found that A-SFE regulated the polarization of TAMs, and inhibited the metastasis and invasion ability of cancer cells. The results indicate that A-SFE could assist anti-CRC in terms of anti-metastasis and regulating TME, which made up for the deficiency of OXA. In addition, it has been reported that Danggui Sini decoction, which mainly consists of *Angelica sinensis*, protected against oxaliplatin-induced peripheral neuropathy (Ding et al., 2020). In conclusion, A-SFE has the potential to become an adjuvant drug for OXA in the treatment of CRC, which provides new strategies for anti-colorectal cancer research.

## Data Availability

The original contributions presented in the study are included in the article/Supplementary Material; further inquiries can be directed to the corresponding author.
