# Supplementary material for: Supercritical fluid extract of Angelica sinensis promotes the anti-colorectal cancer effect of oxaliplatin
Source: Front Pharmacol. 2022 Nov 4;13:1007623. doi: 10.3389/fphar.2022.1007623 (PMC9672077; doi:10.3389/fphar.2022.1007623)
Supplement: Supplementary file 1 [file Table1.DOCX]

Supplementary Material

# Supplementary Tables

Table S1 Tumor volume in each group in vivo (x±*s*) (*n* = 6)

| day | Tumor volume (mm^3)^ | | | |
| --- | --- | --- | --- | --- |
|  | **Model** | **OXA** | **OXA+A-SFEL** | **OXA+A-SFEH** |
| 8 | 256.56 ± 81.33 | 133.20 ± 56.17 | 120.23 ± 43.07 | 71.63 ± 38.44 |
| 9 | 377.12 ± 61.53 | 213.37 ± 54.69 | 191.86 ± 63.80 | 140.43 ± 17.98 |
| 10 | 557.59 ± 142.34 | 310.35 ± 80.48 | 277.32 ± 69.40 | 203.40 ± 36.10 |
| 11 | 663.55 ± 143.04 | 417.86 ± 109.96 | 341.69 ± 77.71 | 257.96 ± 45.62 |
| 12 | 746.83 ± 169.51 | 481.14 ± 118.19 | 422.43 ± 65.85 | 328.01 ± 65.52 |
| 13 | 879.23 ± 165.06 | 572.53 ± 189.14 | 553.72 ± 116.36 | 441.56 ± 89.90 |
| 14 | 1111.75 ± 267.74 | 719.10 ± 205.46 | 658.27 ± 143.95 | 525.62 ± 101.44 |
| 15 | 1631.50 ± 191.46 | 889.49 ± 250.52 | 801.60 ± 197.98 | 640.69 ± 103.90 |

Table S2 Conversion of drug concentration (μg/mL) to crude drug concentration(mg crude drug/mL)

| Name | Unit | 1 | 2 | 3 | 4 | 5 | 6 | 7 | 8 |
| --- | --- | --- | --- | --- | --- | --- | --- | --- | --- |
| A-SFE | μg/mL | 2 | 4 | 8 | 16 | 32 | 64 | 128 | 256 |
|  | mg crude drug/mL | 0.1 | 0.2 | 0.4 | 0.9 | 1.8 | 3.5 | 7.1 | 14.1 |
| Z-ligustilide | μg/mL | 1.5 | 3.0 | 6.1 | 12.2 | 24.4 | 48.7 | 97.4 | 194.8 |
|  | mg crude drug/mL | 0.3 | 0.6 | 1.1 | 2.3 | 4.5 | 9.0 | 18.0 | 36.1 |

## 2. Supplementary Figures


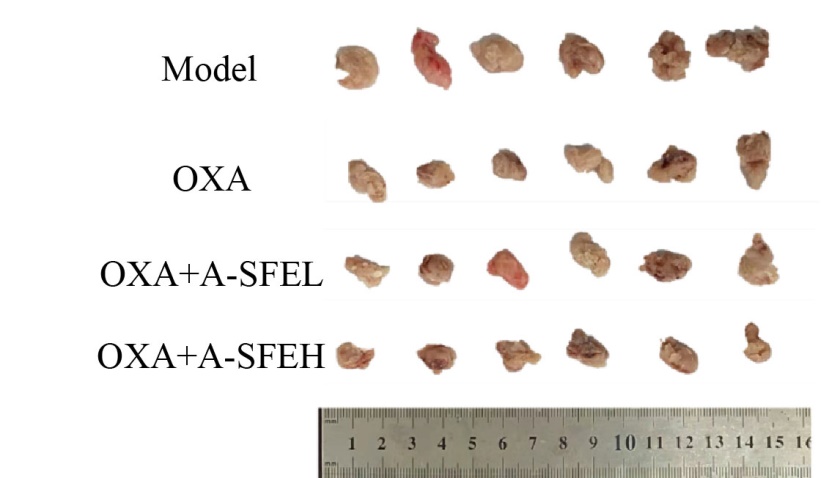


**Figure S1.** Images of tumors in each group
